# “I’d never have that operation again” – a mixed-methods study on how patients react to adverse outcomes following foot and ankle surgery

**DOI:** 10.1186/s13047-022-00590-z

**Published:** 2022-12-09

**Authors:** Israa Abdalla, Aaron P. Robertson, Vivienne Tippett, Tom P. Walsh, Simon R. Platt

**Affiliations:** 1 Faculty of Health, School of Clinical Sciences, Queensland University of Technology (QUT), Kelvin Grove, Queensland 4059 Australia; 2 Department of Orthopaedics, Gold Coast Hospital and Health Service, Southport, Queensland 4215 Australia

**Keywords:** Postoperative complications, Emotions, Orthopedics, Foot, Ankle

## Abstract

**Background:**

Adverse outcomes arising from foot and ankle surgery, including lack of pain relief, increased disability and perioperative complications are infrequent but inevitable. This mixed-methods study aims to explore the impact of adverse outcomes on patients following nonemergent foot and ankle surgery.

**Methods:**

Patients who underwent foot and ankle surgery over a two-year period were invited to participate in this study if they reported an adverse outcome. Qualitative assessment consisted of individual semi-structured interviews, designed to explore the decision they made to have surgery and the impact of the outcome after surgery. Quantitative assessment was performed using questionnaires on demographics, current analgesia, foot pain, health-related quality of life, psychological health, and regret.

**Results:**

Twelve participants (eight women) consented for inclusion in this study. Current foot pain was high in 10 participants, five met the criteria for central sensitisation syndrome and two had clinically significant pain catastrophising. Most participants regretted their decision to have surgery. The three major themes identified were expectations, communication, and alternatives.

**Conclusions:**

Self-reported adverse outcomes following foot and ankle surgery were prevalent and participants in this study consistently complained of persistent pain. Regret was common and reasons cited for their adverse outcomes centred around the feelings of inadequate communication and failure to meet expectations.

**Supplementary Information:**

The online version contains supplementary material available at 10.1186/s13047-022-00590-z.

## Background

Adverse outcomes occur across all surgical disciplines, varying in severity and impact. Some adverse outcomes affect people for short periods e.g., surgical site infection, while others become chronic and may have a greater impact than the original complaint e.g., complex regional pain syndrome. Patients may also consider an adverse outcome to be a lack of relief from the original complaint, particularly if their procedure incurred a long period of postoperative convalescence, or simply did not reach their preoperative expectations. Adverse outcomes can be challenging to manage and should patients feel sufficiently aggrieved, can lead to prolonged and protracted episodes of care, and litigation in some cases. Understanding how patients who sustain adverse outcomes reflect on their decision to have surgery, elective surgery in particular, is not well known but could be important in helping to educate patients preoperatively about the full range of potential outcomes and how these may affect them.

True emergent surgery for orthopaedic foot and ankle complaints is rare. Most surgery for foot and ankle trauma (urgent) can be staged and planned, and involves the patient’s involvement in the decision-making process. Elective surgery in most circumstances provides patients with ample time to consider their options. Surgery for acute trauma generally aims at restoring anatomy and function and the main indication for elective orthopaedic surgery is pain or loss of function, with surgical intervention aimed at reducing pain and restoring function. Patients and foot and ankle surgeons are generally afforded time to make the decision as to whether the benefits surgery outweigh the risk of harms.

Patients and surgeons must balance the risk of adverse outcomes with the potential benefits of surgery, but patients may not fully appreciate how an adverse outcome will impact them and they may not understand the level of risk they are subjecting themselves to because they are in pain. Indeed, people with chronic pain have been reported to have lower executive functioning and perform worse in tasks related to gambling [1], meaning that their process for decision-making may not be optimal. Patients are also unlikely to fully appreciate how an adverse event will affect them until it happens and surgeons can not know exactly how each patient will feel if an adverse outcome is to eventuate. Previously, operative success was measured by assessing clinical complications, readmission and reoperation rates; however, there has been a shift towards examining patient reported outcome measures such as satisfaction (a combination of expectation and the actual outcome) and regret [2, 3].

Sustained pain is more common than many people may appreciate, with unfavourable pain outcomes following total joint arthroplasty at the hip and knee ranging from 7 to 23% and 10–34%, respectively [4]. Moreover, chronic (> 3 months duration), persistent pain is reported in half of patients undergoing all orthopaedic surgery [5] and given pain is the main reason for elective surgery, patients can react to a failure to resolve their symptoms swiftly with hostility and confusion; patients may regret their decision to undergo surgery. This is particularly pertinent with foot and ankle surgery which can take longer than other regions to resolve postoperatively.

Although this has not been well reported in the orthopaedic literature, a systematic review across multiple surgical disciplines, the majority related to cancer, suggests that one in seven patients self-report decisional regret [6]. And while qualitative work on ankle fractures has been previously investigated [7], data specific to a range of foot and ankle surgeries are lacking. Understanding why patients feel aggrieved when they experience adverse outcomes may help improve how patients are informed preoperatively and may enhance the information patients have available to them to make informed preoperative decisions. The aim of this scoping study was to explore the experience of patients with adverse outcomes following foot and ankle surgery.

## Methods

A cross-sectional, mixed-methods design was used for this study. The study was completed in two phases. The first phase (qualitative) was a detailed semi-structured interview discussing the experiences of the participant. The second phase (quantitative) entailed the participants completing the following questionnaires: Manchester-Oxford Foot and Ankle Questionnaire (MOxFQ) [8], Central Sensitization Inventory (CSI) [9], Pain Catastrophizing Scale (PCS) [10], Decision Regret Scale (DRS) [11] and the EuroQol 5-dimensions 5-levels (EQ-5D-5L) [12].

### Participant recruitment

A purposive sampling technique was used for this study. Invitation letters were mailed in between January and February 2020 to all patients who had foot or ankle surgery under the senior author’s clinic (SRP) over a two-year period (January 2017–December 2018) at the Gold Coast Hospital and Health Service (GCHHS). Meaning all patients were a minimum 12-months following their surgery. The invitation letters invited patients who felt that they had a sub-optimal outcome or obtained an adverse outcome to contact the study coordinator (TPW) to assess their eligibility for participation and discuss the study protocol.

### Eligibility criteria

The inclusion criteria included: Adults who have had nonemergent (both urgent and elective were included, so long as the patient provided informed consent) foot or ankle surgery at GCHHS in the past two-years. The exclusion criteria (1) people with a history of cognitive impairment or the inability to understand English, and (2) those who are non-ambulatory. Eligible participants were provided with an information sheet and consent forms and asked to attend a GCHHS site for data collection. Ethics approval to conduct the study was obtained from the GCHHS Human Research Ethics Committee (project ID: 58416).

### Participant characteristics

Participant demographics and characteristics were collected using a questionnaire and included information about their age, sex, body weight and height, and postcode. Weight (kg) was divided by height (m)^2^ to calculate body mass index (BMI). Participants were also asked to report the type of operative procedure performed and their current analgesic use.

### Foot pain and disability

Foot pain and disability was assessed using the MOxFQ [8]. The MOxFQ is a 16-item questionnaire that comprises three separate underlying dimensions: walking/standing problems (seven items), foot pain (five items), and social interaction (four items). Item responses are each scored from 0 to 4, with 4 representing the most severe state. Responses from each item within a category are summed to produce scale scores that can be converted to a metric from 0 to 100 with 100 indicating the most severe state. The three domains and a summary score are all used to describe the sample [8].

### Psychological health (central sensitisation and pain catastrophising)

Central sensitisation has been proposed as a common pathophysiological mechanism to explain related syndromes for which no specific organic cause can be found. The CSI is a two-part questionnaire used to determine if those with pain have central sensitisation syndrome. Only Part A was used for this study, which assesses 25 health-related symptoms common to central sensitisation syndrome, questions are graded via a five-point Likert scale with total scores ranging from 0 to 100. A score of ≥40 in Part A has been found to be clinically significant in identifying those with and without central sensitisation syndrome [9].

Pain catastrophising is defined as an exaggerated negative orientation toward actual or anticipated pain experience [10]. The PCS is a 13-item questionnaire that was developed to facilitate research on the mechanisms by which catastrophising impacts on pain experience. The PCS instructions ask participants to reflect on past painful experiences, and to indicate the degree to which they experienced each of 13 thoughts or feelings when experiencing pain, on a 5-point scale, anchored from 0 (not at all) to 4 (all the time). The PCS yields a total score (0–52) and three subscale scores assessing rumination (0–16), magnification (0–12) and helplessness (0–24), which were used to describe the participants.

### Decision regret

The DRS is a five-item tool used to measure remorse or distress after a healthcare decision [11]. It is a widely validated tool in a range of patient populations. Scores are scaled from 0 to 100 with higher scores demonstrating a higher degree of regret. There are no established thresholds for clinically significant regret.

### Health-related quality of life

The EQ-5D-5L tool was included to measure health-related quality of life (HRQoL) [12]. The EQ-5D-5L includes five dimensions: mobility, self-care, usual activities, pain or discomfort, and anxiety or depression. Within each dimension there are five possible responses or levels participants can report: no problems, slight problems, moderate problems, severe problems, and extreme problems. Responses were transformed into values ranging from 1 to 5, with 5 being extreme difficulty.

### Qualitative data

Face-to-face semi-structured interviews were conducted in a quiet room and audio-recorded to facilitate transcription, coding, and analysis. Interviews were conducted by IA, a female novice undergraduate researcher, under the guidance of VT, an experienced researcher. Following training with VT and piloting with people not associated with the study, IA conducted the semi-structured interview format designed to prompt all participants to share their experiences and perceptions across the full trajectory of their problem i.e.: the events leading up to the procedure, how the decision to undergo surgery was made and the participants view on how information regarding the procedure was presented. IA was not known to the participants at the time of the interviews, but was introduced as an Honours student conducting research with the department who was interested in determining how people feel after experiencing an adverse event. Participants were informed that they would not be identified. The semi-structured interview schedule is provided in Supplementary File 1.

### Data analysis

Quantitative data were analysed with SPSS v26.0 (IBM SPSS Statistics, Armonk, NY, USA). Quantitative data were first analysed descriptively using means, medians, interquartile range (IQR) and standard deviation (SD), to summarise the characteristics of the participants. Transcribed qualitative interview data were coded and subjected to inductive qualitative analysis. Sorting into categories, concepts, and themes, then organised using with NVivo v12.0 (QSR International Pty Ltd.) by IA and VT.

## Results

### Participant characteristics

A total of 154 participants were identified as undergoing foot and ankle surgery during the two-year period studied and were sent a letter of invitation. Twenty-three people responded and expressed interest. Of the 23 people who responded and screened as being eligible, 11 either failed to attend their appointed time or were unavailable on the day of data collection , resulting in a final sample of 12 participants. Eight participants were women, with a mean (SD) age of 60.0 (18.8) years and BMI 27.3 (3.3) kg/m^2^, respectively. A summary of participant characteristics, foot pain, psychological health, HRQoL and decision regret is presented in Table 1.

**Table 1:** Participant characteristics

	Median (IQR)
Age, years^a^	60.0 (18.0)
Sex	8 women, 4 men
BMI, kg/m^2a^	27.3 (3.3)
Duration of foot pain, years	3 (8)
MOxFQ, points	
Walking/standing^a^	76.5 (21.1)
Pain	72.5 (23.8)
Social interaction	75.0 (37.5)
Current analgesia, n	
Nil	2
Paracetamol	6
Non-steroidal anti-inflammatory drugs	0
Opioids	5
Other	0

*BMI* body mass index, *IQR* interquartile range, *kg* kilograms, *m* meters, *MOxFQ* Manchester-Oxford Foot and Ankle Questionnaire

^a^Mean (standard deviation)

### Procedures

A variety of hindfoot, midfoot and forefoot procedures were performed on the participants: Achilles tendon repair (1), ankle arthrodesis (2), triple arthrodesis (2), subtalar joint arthrodesis (1), calcaneal fracture repair (1), tarsometatarsal joint arthrodesis (1), extensor hallucis longus repair (1), hallux valgus correction (1) and hammertoe correction (2).

### Foot pain

Foot pain measured with the MOxFQ was high in both the summary score and the three measured domains. Median (IQR) scores of 78.6 (23.2) points for walking / standing, 72.5 (23.8) points for pain and 75.0 (37.5) points for social interaction were calculated. Three participants were not taking any analgesia, but five were using codeine or other opioids for their foot or ankle pain.

### Psychological status, HRQoL and decision regret

Five participants met the definition of central sensitisation syndrome. The mean (SD) score of the CSI was 37.1 (16.8) points. The median (IQR) PCS score was 8 (19) points. Two participants scored ≥30 points and were classified as having pain catastrophisation. The EQ-5D-5L found that participants reported problems with pain/discomfort most frequently (100%), followed by personal care (92%), usual activities and mobility (both 75%), and anxiety / depression (50%), Table 2. The global mean (SD) for DRS was 47.1 (35.4) points. One participant reported no decision regret (DRS score 0), three reported mild regret (DRS score 5 to 25) and eight reported moderate to strong regret (DRS score ≥ 30).

**Table 2:** Health-related quality of life dimensions, as measured by the EuroQoL-5D-5L

*Dimension*	*Level*				
	No problems	Slight problems	Moderate problems	Severe problems	Extreme problems
Mobility	3	2	5	1	1
Self-care	9	2	1	0	0
Usual activity	3	1	4	1	3
Pain / discomfort	0	4	4	3	1
Anxiety / depression	6	2	2	0	2

Values are *n*

*Abbreviations*: *EuroQol-5-dimensions-5-levels*

### Qualitative analysis

Interviews were conducted with all twelve participants. IA conducted all interviews with VT or with another student researcher present. The duration of time for each interview ranged from 20 minutes to 60 minutes. Three major themes emerged, with data saturation, from analysis of these transcriptions and are described below. Participant quotes indicative of experiences included in each theme are provided.

#### Communication

The majority of participants believed communication was lacking between the surgical team and the patient throughout their operative journey including postoperatively.

‘And there wasn't a lot of explanation but I was grateful to be having something done. But after I got out of my Achilles boot, he did say to me, just be careful and it may never come right.’ (Participant 3)

Participants reported not being told what to expect from the procedure; the specific risks associated with the procedure; or what changes may need to be implemented after the procedure.

‘They just said it would - yeah, the risks that you were given were any risks that would have happened if you had an operation.’ (Participant 5)

The small number of participants who did not report any issues with communication, reported that they had felt comfortable asking questions as they had operative procedures done in the past. These participants also felt they had enough time to do their own research between consent and the procedure. Several participants believed that written information should be provided to them preoperatively about what the procedure/s involves, any changes that needed to be made including care/support, and what to do if they experienced pain. Better communication during transfer of care from specialist to their General Practitioner (GP) or other health professionals was also identified.

#### Expectations

Half of the participants were unable to recall conversations surrounding expectations of outcomes in the preoperative period.

‘I didn't know what to expect, because it was only when I was sitting on the bed waiting to be wheeled into the theatre - and then the guy saying to me, look, we're going to put these wires through your legs and you're going to have - it just all hit me. I didn't know what he was talking about. I'd never had external fixations before…That's why I went through it, but I didn't expect what's happened. If I'd have known that now I'd never have gone through it. I'd never have that operation again. It's just I didn't think it was going to be that dramatic.’ (Participant 9)

When the results of the procedure were reported to be worse than before the procedure or less than the expected outcome described to them by the surgical team preoperatively, participants expressed their regret at proceeding with the procedure.

‘Coming out of that surgery, I was – my expectation was no pain, which I was told I would have no pain. So no pain whatsoever, and a limp because I would lose the flexibility in my foot. So that’s what I was told was to be the outcome – that’s what we were after. So just I didn’t care about having a [limp], but I was to have no pain.’ (Participant 10)

#### Alternatives

The majority of participants reported that the decision to undergo surgery was theirs, not the surgeons. However, patients also reported that the preoperative information they received from their surgeon influenced their decision to opt for surgery.

‘Yeah. Well, I think he did discuss other options. I mean I think - I can’t remember now but I think it was put to me we could leave it and see. I think that was one of the options. I can’t, I honestly can’t remember is the truth. I feel pretty sure that other options were discussed and it was known that this was pretty much a last resort.’ (Participant 11)

Although pain scores reported preoperatively varied, some participants felt that only operative interventions were presented to them.

‘Yes, and I thought it would be the only option, and the first time would fix it. But things happen.’ (Participant 1)

The most commonly reported decision point for participants was deciding whether they could ‘live with their pain’ or not. With hindsight, some participants believed they would rather have lived with the pain rather than proceeding with surgery.

‘It is the only option for me... It was. Or I thought it was, but after what I’ve gone through, I think I would have put up with the arthritis.’ (Participant 10)

More than half of the participants failed to recall any treatment options other than surgery being presented to them.

The majority of men reported problems across all three themes in their responses: expectations, communication and alternatives while less than half of the women touched on all three themes in their interviews. Most participants reporting themes relating to expectations, communication, and alternatives also reported they were taking analgesics, with codeine being the most common medicine used at the time of data collection. With respect to decisional regret, most participants with high decisional regret reported complaints across all three themes. The one participant with no decisional regret reported they were felt they were told what to expect and were satisfied by the communication level however they felt they were not given alternatives to surgery. Most people who reported they were presented with alternatives to surgery still reported a high decisional regret with one participant reporting mild decisional regret. All participants with severe and extreme central sensitisation were not satisfied with the level of communication and felt that they were not completely informed about what to expect from surgery.

### Improving the process

Participants frequently suggested that the postoperative process would be improved if written information was provided regarding their procedure, what was found during the procedure, what they should be expecting in the weeks following the surgery, and what recovery may look like for them. Several participants would have liked to be more rigorously assessed for their suitability for surgery such as assessing their mental health, support network and understanding of the process. Opportunities during the preoperative consultation to discuss what the next steps would be if the procedure failed was also raised.

## Discussion

This study is the first to undertake a mixed-methods analysis to explore patients’ feelings and experiences following an adverse outcome after a range of foot and ankle surgeries. It found that the majority of patients who feel that they have sustained an adverse outcome report disabling levels of persistent foot and ankle pain. Patients feel that a range of factors may have led to their adverse outcome, but these are generally directed towards the care that they received. They often regret their decision to have surgery, feel that patient-doctor communication and management of expectations could be improved, and they do not recall being provided with alternative care options.

Participants reflected that the absence, or delivery, of information presented to them during their preoperative consultation(s) had a negative impact on their postoperative recovery. They frequently referenced that the lack of continuity of care contributed to their poor outcome. Being a public-sector, teaching-hospital service, it is highly likely and inevitable that patients will see a range of clinical staff throughout their surgical journey. Furthermore, this service is high-volume and manages complex elective and trauma surgery from across the region. The introduction of decision-aid tools could help (a) standardise the patient experience, (b) engage the patient and surgical team and (c) may be especially useful for surgical disciplines, where training in the shared decision-making process may be lacking [13]. Critical communication points often recognised by the participants included the transfer of care and postoperative consultations [14]. Participants noted that verbal and written information would have been beneficial between surgeon and patient, surgeon and GP, and operative team and other health services.

The issues surrounding communication for patients may be symptomatic of the structure of the Australian public health system: The high number of patients allocated to surgical clinics results in brief consultations. There is also heterogeneity between doctors regarding their level of training and experience for managing musculoskeletal foot and ankle complaints. This situation is further compounded by the fact that foot and ankle orthopaedic patients often have symptoms for years prior to referral for surgery in the public sector, and routinely do not access allied health for the provision of non-surgical care prior to referral [15]. Patients may feel it necessary to make a rapid decision to have surgery, without fully appreciating the potential benefits and harms, as they do not want to be returned to a waiting-list. Furthermore, patients suffering with chronic pain may also prefer surgical interventions, perceiving them to be more aggressive and thus, more impactful. These patients may therefore benefit from both comprehensive triaging or screening clinics prior to their orthopaedic consultation where they could be afforded more time and education before meeting with surgeons.

The participants in our study reported that the information presented to them was often in communication poor environments, with limited time to consider their treatment options, which may have contributed to an increase in regret levels [14]. It is crucial to understand the consequences and impact of not communicating important information on the patient’s decision and feelings [14]. The readability of information sheets and the consent form is also an important aspect of care for these patients. A recent study from our unit also found that found that whilst a selection of foot and ankle procedural information sheets were below the recommended reading age (between the 6-8th grade level), the consent form was above the recommended reading age [16] – meaning that some patients may not entirely understand what they are consenting to.

Unsurprisingly, regret was prevalent in this group, especially amongst those with persistent pain. Regret is associated with future decision-making and can influence future behaviours [14]. However, during the interviews, the participants suggested that they were still open to further surgical procedure(s) as a means of solving their adverse outcomes regardless of what has occurred in the past. The willingness to undertake another operation, even despite regret on the original procedure, may reflect the impact of chronic pain has on decision-making and the desperate desire for resolution. Their confidence in surgery as a treatment option remained, but they would request more communication.

Overall, these findings are in accordance with previous research which found that patient satisfaction can be achieved if preoperative expectations have been met and satisfactory pain relief is achieved for patients undergoing lower-limb arthroplasty [17]. Moreover, modifiable factors such as communication and the provision of tailored information can increase patient satisfaction scores [18]. Interestingly, other studies have shown that patient satisfaction was independent of the rate of complications and suggested poor surgeon communication can lead to increased dissatisfaction regardless of the presence of complications [19]. Evidence elsewhere highlights the need for considering patient preferences when making the decision for surgery, such as the need for postoperative support which may lead to lower levels of regret [20]. Identifying patients more likely to report decision regret or complain of poor outcomes, preoperatively, would clearly be worthy of further study.

It is important that the results of this study be considered in light of its limitations. Firstly, it is important to note that this study has focused only on patients’ personal reflections and not on surgeons’ experiences and feelings following adverse outcomes. Views on improving communication are important throughout the entire process of operative care and by only including people who have had an adverse outcome, their reflection on the remembered events as opposed to the actual events may be clouded. Secondly, the single-centre design may have been a limitation, as patients from different hospitals networks and services may have different experiences with adverse effects from foot and ankle surgery. Thirdly, qualitative data is also subject to recall bias, where participants were asked to report on their past exposure which may be subject to omissions or external influences that may compromise the accuracy.

The study has several strengths. To our knowledge this is the first report to explore the patients’ perspective in foot and ankle orthopaedic surgery setting. The findings of this study suggest adverse outcomes following foot and ankle surgery place a heavy burden on patients, and when asked to reflect on their experiences a key theme is one of communication. How surgeons deliver this communication and obtain informed consent, especially in the public-sector where continuity with a practitioner is not guaranteed may require further thought, and may be improved through the use of digital resources [21]. The study highlights that when reflecting on their experiences, patients consider (mis)communication or misunderstanding to be a decisive factor in whether they are prepared for surgery, but even in the face of perceived inadequate communication, patients still underwent surgery. Interestingly, the conduct of the study also resulted in some participants voicing positive feelings about participating – specifically, that they were able to express their emotions about their experiences, which raised the possibility that the interview had some therapeutic effect. Future enquiry, via a clinical trial, may indeed determine if this could be an effective therapy. Collectively, the results provide evidence for the importance of effective communication throughout the operative process.

Adverse outcomes can occur following surgery, but surgical teams should look to make improvements. This study adds to a growing body of research highlighting the importance of patient-doctor communication, ensuring patients are clearly aware of what they should expect during their episode of care, and what the alternatives to surgery are. Future research could investigate the effect of implementing strategies e.g. digital consent that improve communication with patients during the preoperative period. Furthermore, surgeons and patients discuss risk (the chance of something happening) in the preoperative period but perhaps a more meaningful and enlightening area of research is to determine what an adverse outcome e.g. deep infection or non-union, or lack of improvement e.g. persistent pain, actually means to someone, and the impact it has on their life. This would enable a more meaningful discussion between surgeons and patients as they make the decision of whether they are prepared for an operation. Interestingly, no participant questioned the surgeons’ skills or technical competence, and while surgical error should always be considered, these participants focussed their attention on the care they received outside of the operating theatre. Research into how surgeons may identify and educate patients at risk of adverse outcomes following foot and ankle surgeons should be considered.

In conclusion, surgeons and patients may benefit from strategies designed to minimise time stressors, ensure they provide timely and easy to understand instructions, and present clear alternatives and advice regarding expectations. Importantly, other than the provision of more time, ways to improve the patient experience may be low- or no-cost to the health service.

## Supplementary Information

**Additional file 1: Table S1.** Semi-structured interview guide.

## Data Availability

The data which support the findings of this study are available from the corresponding author upon reasonable request.

## Abbreviations

5D-5L: 5 Dimensions-5 Levels
BMI: Body mass index
CSI: Central Sensitization Inventory
DRS: Decision Regret Scale
EQ: EuroQoL
GCHHS: Gold Coast Hospital and Health Service
GP: General Practitioner
HRQoL: Health-related quality of life
IBM: International Business Machines
IQR: Interquartile range
MOxFQ: Manchester-Oxford Foot and Ankle Questionnaire
PCS: Pain Catastrophizing Scale
SD: Standard deviation
