# Supplementary material for: “I’d never have that operation again” – a mixed-methods study on how patients react to adverse outcomes following foot and ankle surgery
Source: J Foot Ankle Res. 2022 Dec 9;15:85. doi: 10.1186/s13047-022-00590-z (PMC9733247; doi:10.1186/s13047-022-00590-z)
Supplement: Supplementary file 1 — Additional file 1: Table S1. Semi-structured interview guide. [file 13047_2022_590_MOESM1_ESM.docx]

**Table S1: Semi-structured interview guide**

1. What type of surgery did you have performed?
2. What outcome/s did you perceive to be below expected?
3. What was your pain or problem like before the surgery? How was it affecting your life?
4. What made you decide to consider and undertake surgery?
5. How do you feel about the potential risks when the doctor/s explained them to you? Do you remember these conversations well (about risk)?
6. Were there any risks you were worried about before the surgery? If so, which ones and why do you think you thought about these?
7. How did you feel about the advice you had been given regarding things you needed to do during your recovery?
8. What were your expectations regarding your expected outcome of the surgery?
9. When did you expect to be recovered from your surgery?
10. What did you expect your pain or function to be like after your surgery had healed?
11. When did you think something wasn’t quite right after your surgery?
12. Did you return immediately to the orthopaedic department, or did you seek care elsewhere e.g. your GP beforehand? How do you think this was handled?
13. Would you have the surgery you had, or another surgery again? Why?
14. Do you consider yourself a lucky person?

**If the participant is still suffering with the adverse outcome**

1. Do you expect your adverse outcome to ever get better? If so, when?
2. Is there anything else you would like to add?
